# Synthesis of fluorescent Molecularly Imprinted Polymer Nanoparticles Sensing Small Neurotransmitters with High Selectivity Using Immobilized Templates with Regulated Surface Density

**DOI:** 10.3390/nano13010212

**Published:** 2023-01-03

**Authors:** Yasuo Yoshimi, Yuto Katsumata, Naoya Osawa, Neo Ogishita, Ryota Kadoya

**Affiliations:** Department of Applied Chemistry, Shibaura Institute of Technology, 3-7-5 Toyosu, Koto-Ku, Tokyo 135-8548, Japan

**Keywords:** molecularly imprinted polymer (MIP), fluorescence, solid phase synthesis, acetylcholine, serotonin, dopamine

## Abstract

To develop nanosensors to probe neurotransmitters, we synthesized fluorescent-functionalized molecularly imprinted polymeric nanoparticles (fMIP-NPs) using monoamine neurotransmitters (serotonin and dopamine) immobilized on glass beads as templates. The size and fluorescence intensity of the fMIP-NPs synthesized with blended silane couplers increased with the presence of the target but were insensitive to the target analogs (L-tryptophan and L-dopa, respectively). However, when the template is anchored by a pure silane agent, both the fluorescence intensity and particle size of the fMIP-NPs were sensitive to the structural analog of the template. Another fMIP-NP was synthesized in the presence of poly([2-(methacryloyloxy)ethyl] trimethylammonium chloride (METMAC)-*co*-methacrylamide) grafted onto glass beads as a dummy template for acetylcholine. Acetylcholine increased the diameter and fluorescence intensity of the fMIP-NP, but choline had no effect. When the homopolymer of METMAC was used as a template, the fluorescence intensity and size of the resulting nanoparticles were not responsive to either acetylcholine or choline. The principle of increased fluorescence intensity due to specific interaction with the target substance is probably due to the increased distance between the fluorescent functional groups and decreased self-quenching due to the swelling caused by the specific interaction with the template. The results also indicate that MIP nanoparticles prepared by solid-phase synthesis can be used for targeting small molecules, such as the neurotransmitters addressed in this study, by adjusting the surface density of the template.

## 1. Introduction

In the animal brain, neurons perform a variety of complicated transduction operations in parallel, including the processing of emotions, learning, and judgment. Neurotransmitter release primarily serves to improve communication between neural terminals [1]. Studying how the brain processes information requires an appreciation for how and when neurotransmitters are secreted. Neurotransmitters are detected by either an amperometric method [2,3,4] or a microdialysis method [5,6,7,8,9,10,11,12]. The former method uses a microfiber electrode inserted into the brain to detect the redox current of the transmitters. The latter uses a hollow fiber membrane inserted into the brain to collect the transmitter and pump it into the detector of the transmitters. However, the former method has low selectivity, and the latter method has the disadvantage that real-time data cannot be obtained. Therefore, these analysis methods are performed in parallel, and the data are integrated to infer the behavior of the neurotransmitters. However, even if the transmitter involved in the activity can be identified, it is difficult to obtain the exact location and timing of secretion. If a probe that converts specific interactions with neurotransmitters into optical signals was available, it would be possible to analyze animal neural activity with high efficiency by staining neurons with the probe and analyzing the detected microscopic images.

There have been sustained attempts to develop imaging techniques to visualize neurotransmitter secretion. For example, Hirose and his colleagues genetically engineered a receptor protein for transmitters, such as glutamate, to hybridize with a fluorescent protein and created a probe that changes fluorescence intensity in response to the transmitter [13]. Imaging of the transmitter was made possible by binding this probe to the neuronal cell membrane. Subsequently, several researchers have attempted to develop proteins that can function as probes using genome editing techniques [14,15,16,17,18,19,20,21]. The use of protein probes to facilitate transmitter secretion, however, has met with limited success. The availability of sufficient probes for nerve staining is a significant factor in this delay, as it can take months to secure them.

A molecularly imprinted polymer (MIP) is a polymer that has been processed using the molecular imprinting technique, which leaves cavities in the polymer matrix with an affinity for a chosen “template” molecule [22]. The MIP can rebind specifically with the target molecule used as the template through the imprinted cavities. The process usually involves initiating the polymerization of monomers in the presence of a template molecule that is extracted afterward, leaving behind complementary cavities. MIPs have the benefit of being simpler and less expensive to design and manufacture than protein receptors. However, a simple method for transducing the precise binding event into an optical signal must be established. We previously discovered a phenomenon in which an MIP nanoparticle increased in size in response to specific interactions with the target [23].

This work involves the development of MIP nanoparticles that contain fluorescent functional groups (fluorescent MIP-nanoparticle: fMIP-NP) in anticipation that the fluorescence intensity will reflect the swelling associated with the specific interactions. We used the “solid-phase molecular imprinting” approach established by Piletsky and his colleagues, which involves immobilizing templates on the surface of glass beads and removing the template from the MIP after synthesis. [24]. This method can easily and quickly produce nanoparticles of MIP. The fMIP-NPs were made by immobilizing monoamine neurotransmitters (serotonin and dopamine) in glutaraldehyde and a mixture of amino silanes. Acetylcholine lacks the necessary group for covalent attachment to the glass surface. To mimic acetylcholine, we employed a dummy template made from [2-(methacryloyloxy)ethyl] trimethylammonium chloride (METMAC) copolymerized with methacrylamide (MAAm) grafting on the surface of a glass bead. These templates were used to make the fMIP-NPs. However, the solid-phase molecular imprinting has mainly focused on macromolecular materials, and there are few examples of imprinting on small molecular weight materials such as these neurotransmitters. We present the preparation of a highly selective fMIP-NP for its target neurotransmitters, along with the design of the template immobilization.

## 2. Materials and Methods

**Figure 1:**
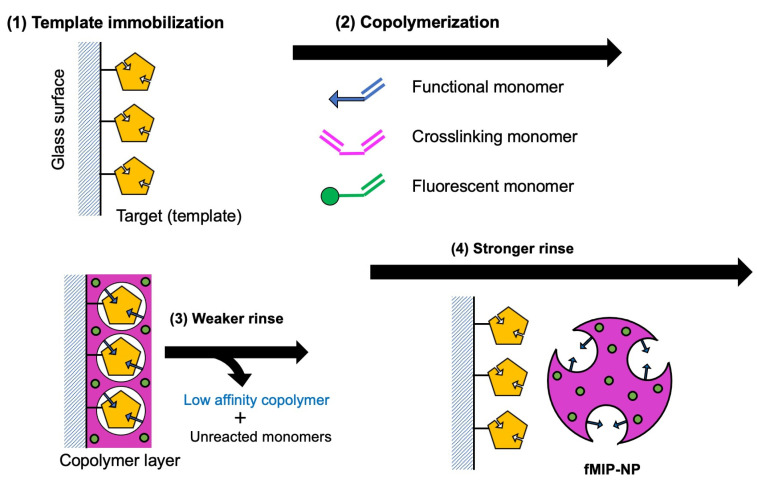
Scheme of the procedure for fMIP synthesis: (1) the target molecule was immobilized on the surface of glass as a template of the MIP, (2) functional monomer, crosslinking monomer, and fluorescent monomer were copolymerized at the vicinity of the surface, (3) copolymer which has low affinity with the template and unreacted monomers was removed by the weaker rinse, and (4) fMIP-NP which has high affinity with the template was dissociated from the surface by the stronger rinse.

The fMIP-NP was prepared using the procedure illustrated in Figure 1. The general processes for the preparation involve (1) immobilization of the template (or dummy template) on the glass beads surface, (2) copolymerization of the functional, crosslinking, and fluorescent monomers, (3) weak washing to remove unreacted monomers and weakly adsorbed polymers, and (4) a strong wash to dissociate the fMIP-NP from the glass surface. 

### 2.1. Chemicals

Serotonin (5-hydroxytryptamine: 5-HT) hydrochloride, L-tryptophan (Trp), dopamine chloride, 3-(3,4-dihydroxyphenyl)-L-alanine (DOPA), *N,N*-diethyldithiocarbamate, methacrylamide, methacrylic acid (MAA), ethyl glycol dimethacrylate (EDMA), and 25% glutaraldehyde aqueous solution were purchased from Fujifilm Wako Chemical Industries Co., Ltd. (Osaka, Japan).

Benzyldeithyldihiocarbamate (BDDC), acetylcholine chloride, choline chloride, 3-methacrylamidophenylboronic acid (MAPBA), 3-aminopropyltrimethoxysilane (APTMS), 3-(2-aminoethylamino) propyltrimethoxysilane (AEAPTMS), propyltrimethoxysilane (PTMS), and 4-chloromethyl benzoic acid were purchased from Tokyo Chemical Industries (Tokyo, Japan). METMAC was purchased from Sigma-Aldrich Co., Ltd. (St. Louis, MO, USA). Water-soluble carbodiimide (WSC) (or 1-ethyl-3-(3-dimethyl aminopropyl) carbodiimide hydrochloride) was purchased from Dojindo Laboratories (Kumamoto, Japan). Glass beads of 50 µm in diameter (Rolloblast^®^) were purchased from Renfert Co., Ltd. (Hilzingen, B-W, Germany). Diallyl fluoresceine (DAF), which is a fluorescent monomer, was synthesized using the procedure described by Liu et al. [25].

### 2.2. Pretreatment of Glass Beads for the Template Immobilization

First, 50 g of the glass beads were boiled in a 1M aqueous solution of sodium hydroxide for 10 min to clean their surface. After washing with 1000 mL of distilled water with a suction glass filter, the beads were dried in an oven (60 °C) overnight. Next, 25 g of these treated glass beads were soaked in a 2 wt.% toluene solution of silane couplers (APTES, AEAPTMS, PTMS, or a mixture thereof) for 24 h to introduce a regulated density of amino groups at their surface. The glass beads were washed with 500 mL of acetone and dried in the oven overnight.

### 2.3. Immobilization of Monoamine Template on Glass Beads

First, 60 mL of a 25% glutaraldehyde (GA) aqueous solution was diluted with 60 mL of phosphate buffer saline (PBS) of pH 7.4 and heated to 80 °C. To this, the aminated glass beads were soaked to replace the primary amino group with the aldehyde group. After 4 h of soaking, the glass beads were washed with 2000 mL distilled water and dried in an oven (60 °C) overnight. Next, 10 g of these GA-immobilized glass beads were stirred in 5 mM of 5-HT or dopamine solution buffered at pH 7.4 by phosphate for 24 h. During this process, nitrogen was continuously bubbled into the solution. Finally, the glass beads were again washed with distilled water (2000 mL) and dried in vacuo for 24 h.

### 2.4. Synthesis of fMIP-NP for 5-HT

First, 3 g of 5-HT-immobilized glass beads, BDDC 0.15 g (0.62 mmol), EDMA 1.29 g (6.49 mmol), MAA 0.36 g (4.2 mmol), DAF 10 mg (0.24 mmol) distilled water (900 µL), and DMF (6 mL) were mixed in a quartz tube (60 mm inner diameter). After pre-bubbling with nitrogen for 20 min for de-oxygenation, the glass-bead suspension was irradiated using Xe-lump (LC-8, Hamamatsu Photonics Inc., Hamamatsu, Japan) through the attached optical fiber with continued bubbling for 25 min. The treated beads were washed with a mixture of 75 mL of DMF and 25 mL of distilled water using suction filtration. The washed beads were packed in a 20 mL column with frit (Spelco Inc., Bellefonte, PA, USA), and 50 mL of DMF, at 60 °C, was pushed through the packed beads to collect the synthesized polymer using air with the help of a 50 mL syringe. The fluid was transferred into a round-bottom flask, and DMF was removed using a rotary evaporator in a vacuum. Next, 100 mL of PBS was transferred (pH 7.4) into the flask to allow the synthesized polymer nanoparticles or fMIP-NP to disperse into the buffer solution as colloidal particles. Another dispersion of the fluorescent non-imprinted polymer nanoparticles (fNIP-NP) was prepared using the same procedure as for the fMIP-NP dispersion except for using the aldehyde-introduced glass beads instead of the serotonin-immobilized beads.

### 2.5. Synthesis of fMIP-NP for Dopamine

The dopamine-immobilized glass beads 5 g, MAPBA 0.25 g (1.22 mmol), MAA 0.105 (1.22 mmol), EDMA 2500 g (12.6 mmol), DAF 10 mg (0.24 mmol), BDDC 0.15 g (0.62 mmol), DMF (6 mL), and distilled water (900 μL) were mixed in a quartz crystal test tube. The fluid was deoxygenated using nitrogen-bubbling for 20 min. Light from the xenon lamp, as described above, was irradiated into the fluidized glass beads with vigorous N_2_-bubbling for 25 min for copolymerization of EDMA, MAA, MAPBA, and DAF. The beads were washed with a 100 mL mixture of distilled water and DMF (1:4 *v*/*v*). The washed beads were transferred into a 60 mL column with frit, and 50 mL of DMF (at room temperature) was pushed through the beads to allow the copolymer to dissociate from the beads. The copolymer dispersion was transferred into a round-bottom flask, and DMF was removed using evaporation. Then, 100 mL of PBS was poured into the flask and shaken vigorously to allow the copolymer to disperse in the PBS (pH 7.4) as fMIP-NP of dopamine. An fNIP-NP was also prepared by the same procedure as the fMIP-NP dispersion, except for using the aldehyde-introduced glass beads instead of the dopamine-immobilized beads.

### 2.6. Graft Copolymerization of METMAC as a Dummy Template of Acetylcholine 

**Figure 2:**
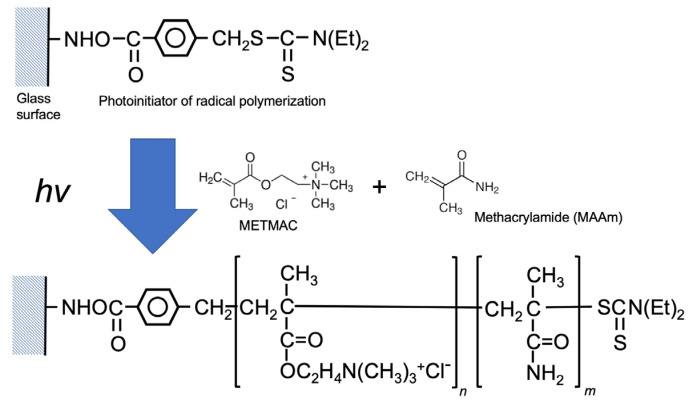
Graft copolymerization of METMAC and MAAm on the surface of the glass beads as the dummy template for acetylcholine.

Acetylcholine does not have a proper functional group for binding covalently with glass surfaces. METMAC has a structure very similar to that of acetylcholine chloride, thus poly (METMAC-*co*-MAAm) grafted on the surface was used as a dummy template for preparation of the acetylcholine imprinted nanoparticle as shown in Figure 2.

First, 10 g of the glass beads aminated by treatment with APTMS were stirred in 20 mL of DMF solution, including 0.2 M WSC and 0.1 M *p*-chloromethyl benzoic acid, for 24 h. The beads were washed with 500 mL of methanol and 2000 mL of distilled water and dried in the oven overnight. The chloromethylated beads were stirred in 20 mL of an ethanolic solution of 0.8 M sodium diethyldithiocarbamate to introduce the radical polymerization initiator (diethyldithiocarbamate benzyl group) onto the surface of the glass beads. Finally, the initiator-coated glass beads were washed with 500 mL of methanol and 2000 mL of water, dried in a vacuum, and stored in the fridge before use for graft polymerization.

METMAC was recrystallized in acetone before the polymerization. METMAC and MAAm (0.95 g in total) were solved in a mixed solvent of DMF (18 mL) and distilled water (8 mL). Then, 5.0 g of the initiator-coated glass beads were fluidized in the solution using nitrogen bubbling for 15 min for deoxidization in the quartz tube. Next, the fluidization was continued under irradiation by the xenon lamp LC8 to graft the copolymer of METMAC and MAAm on the glass beads for 15 min. The beads were washed with a mixture of DMF 75 mL and DW 25 mL, and were dried in vacuo.

### 2.7. Preparation of the fMIP-NP for Acetylcholine

The grafted glass beads (3.0 g), MAA 360 mg (4.18 mmol), EDMA 1.29 g (6.51 g), DAF 10 mg (0.24 mmol), BDDC 0.15 g (0.62 mmol), DMF (6 mL), and distilled water (900 μL) were mixed in a quartz test tube. The fluid was deoxygenated using nitrogen bubbling for 20 min. Light from the LC8 xenon lamp described above was irradiated into the fluidized glass beads with vigorous N_2_ bubbling for 25 min for copolymerization of EDMA, MAA, and DAF. The beads were packed in the 20 mL column with frit (Spelco Inc., Bellefonte, PA, USA). Next, 25, 50, and 75% DMF aqueous solutions (100 mL each) were pushed through the packed column, sequentially, for washing the beads. Finally, DMF was pushed through the beads to collect the fMIP-NP imprinted with the MEMTAC copolymer as a dummy template of acetylcholine. The fluid was transferred to a round-bottom flask, and DMF was removed by rotating the evaporator from the fluid, and the flask was dried in vacuo for 24 h. The fMIP-NP was redispersed in 100 mL PBS (pH 7.4).

### 2.8. Evaluation of the Sensitivity and Selectivity of the fMIP-NP

The prepared dispersion of fMIP-NP (or fNIP-NP in the PBS) was mixed with a solution of the target molecules or its analogs in the PBS with a volume ratio of 3:7. The fluorescent intensity at 525 nm with an excitation light of 425 nm was measured with a fluorometer FP-750 (JASCO, Hachioji, Japan). The radius of fMIP-NP or fNIP-NP was measured using light scattering spectroscopy with DelsaMax Pro (Beckman Coulter, Brea, CA, USA). The dependencies of the fluorescent intensity and radius of the fMIP-NP on the concentration of the target molecule used as the template and their analogs were evaluated. All measurements were performed at room temperature.

## 3. Results

### 3.1. Sensitivity of fMIP-NP of 5-HT

**Figure 3:**
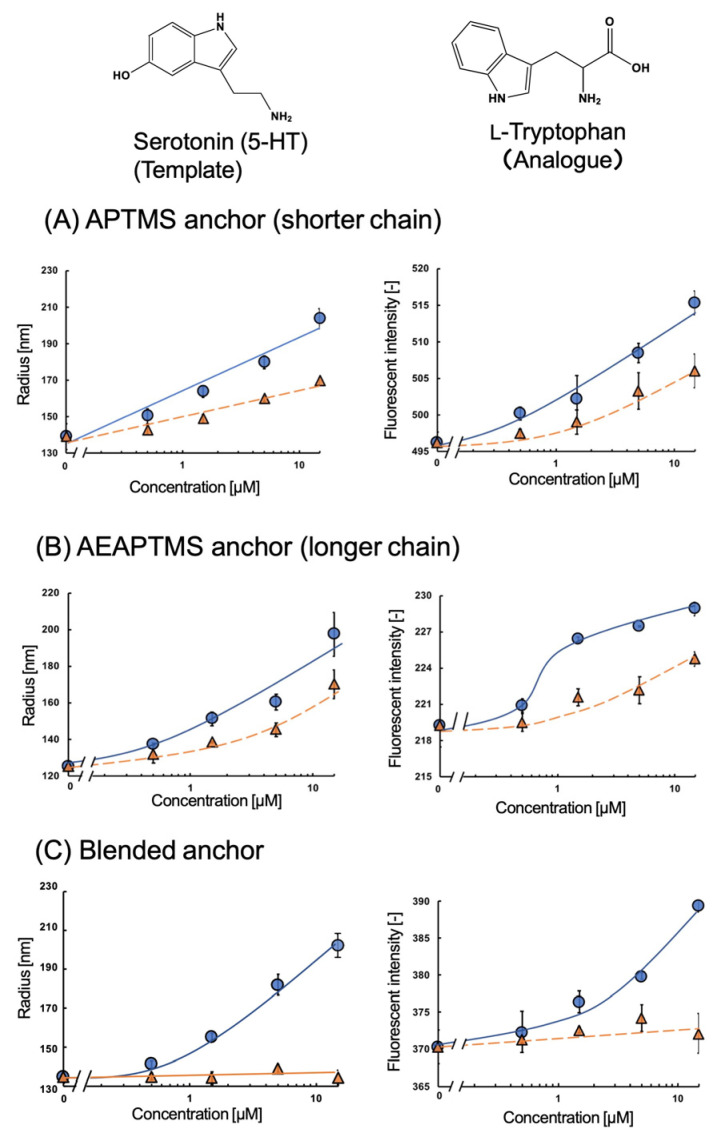
Effect of the 5-HT (circles) and Trp (triangles) on the radius (**left**) and fluorescent intensity (**right**) of the fMIP-NP synthesized with the 5-HT as a template anchored with (**A**) APTMS, (**B**) AEAPTMS, or (**C**) their 1:1 blend in mole.

**Figure 4:**
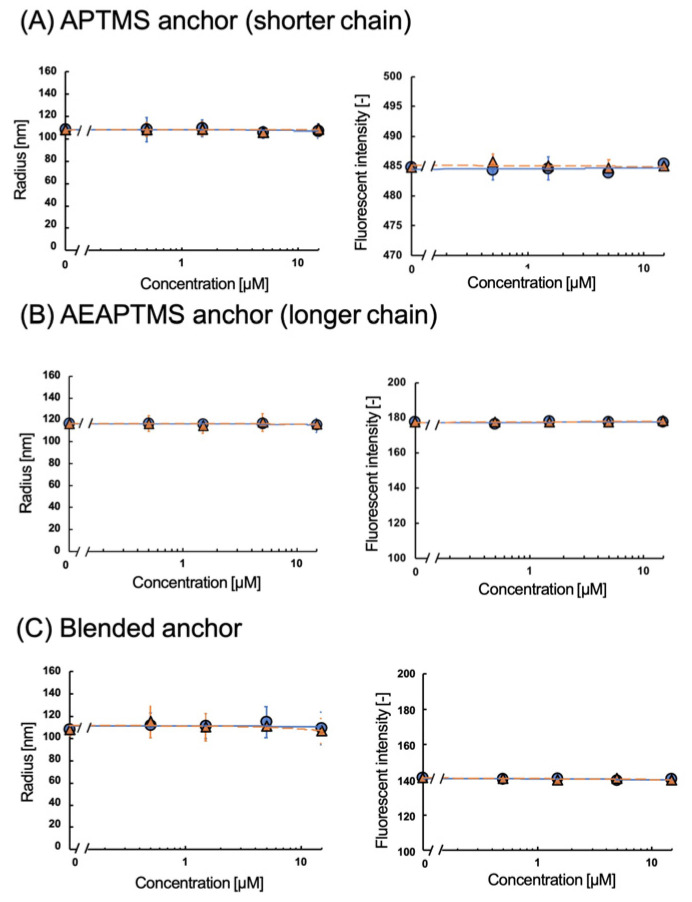
Effect of the 5−HT (circles) and Trp (triangles) on the radius (**left**) and fluorescent intensity (**right**) of the fNIP-NP synthesized at the glass beads coated with (**A**) APTMS, (**B**) AEAPTMS, or (**C**) their blend 1:1.

The fMIP-NPs were synthesized using a 5-HT template immobilized on the surface of glass beads via APTMS, AEAPTMS, or their 1:1 molar mixture. The dependency of the fluorescent intensity and average radius of the fMIP-NP on the concentrations of 5-HT and Trp are shown in Figure 3. The increase in 5-HT or Trp concentration increased the fluorescent intensity and the radius of the fMIP-NP using pure APTMS or AEAPTMS as anchors for the template immobilization. However, the radius and the fluorescent intensity of the fMIP-NP using the blended silanes increased with the increase in the 5-HT concentration but were insensitive to the Trp concentration. It indicates that blending of the silane coupler is effective for the synthesis of highly selective fMIP-NP. The radius and fluorescent intensity of the fNIP-NP, which is synthesized without the template, were insensitive to both 5-HT and Trp, as shown in Figure 4. The results indicate that the fMIP-NP increases the size and fluorescent intensity through the interaction between the imprinted cavity and analytes (5-HT or Trp).

**Figure 5:**
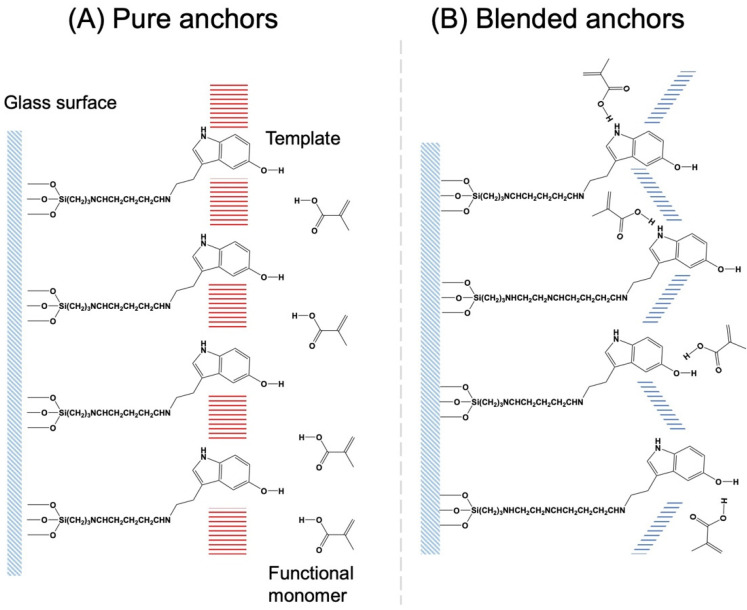
The templates anchored with the pure anchors have strong interactions with their neighbors, so the interaction with the functional monomer is weaker (**A**). However, the template anchored with the blended anchors have weaker interactions with their neighbors due to misalignment, so the interaction with the functional monomer is stronger (**B**).

The quality of the imprinted cavity is most likely affected by the condition of the template immobilized by the silane coupler. The MIP’s specificity is often governed by the way the functional monomer interacts with the template. If the template is fixed to the solid surface by means of a pure anchor, then each molecule of the template will be at the same distance from the surface. In this scenario, as depicted in Figure 5, the template molecules would interact strongly with one another, therefore organizing the self-assembled monolayer of the template and attenuating the interaction between the functional monomer and template. When silane agents with differing alkyl lengths are combined, the template positions are misaligned, and the interaction between the templates is weaker. The resulting stronger interactions with functional monomers may lead to the formation of persistent imprinted cavities and the synthesis of highly selective fMIP-NPs.

### 3.2. Sensitivity of fMIP-NP of Dopamine

**Figure 6:**
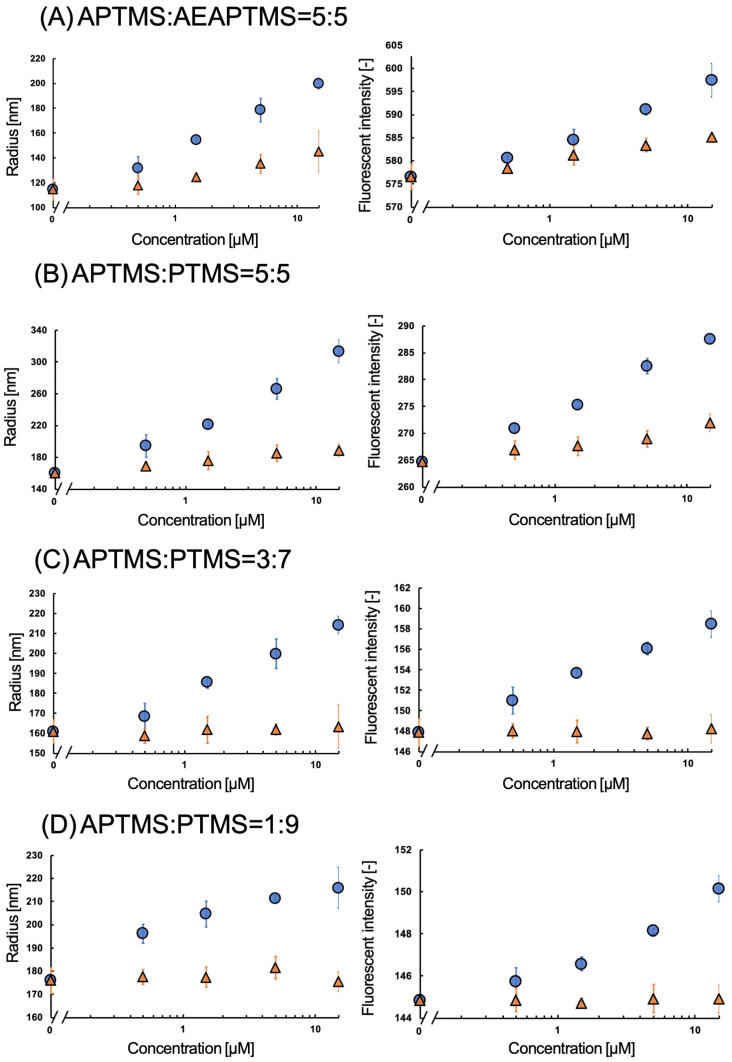
Effect of the dopamine (circles) and DOPA (triangles) on the radius (**left**) and fluorescent intensity (**right**) of the fMIP-NP synthesized with the 5-HT as a template anchored with a mixture of (**A**) APTMS and AEAPTMS (1:1 in mole) or PTTMS and PTMS, (**B**) 5:5, (**C**) 3:7, and (**D**) 1:9 in mole.

**Figure 7:**
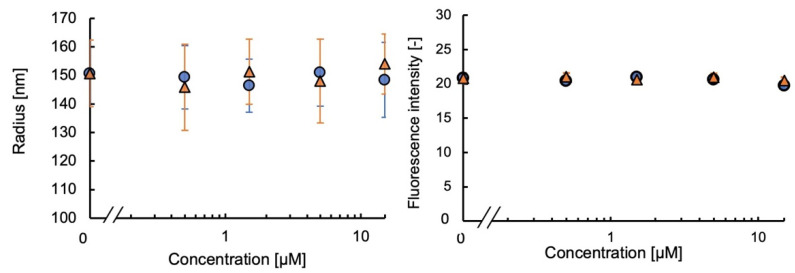
Effect of the dopamine (circles) and DOPA (triangles) on the radius (**left**) and fluorescent intensity (**right**) of the fNIP-NP synthesized with the dopamine template anchored with a mixture of APTMS and PTMS (3:7 in mole).

The fMIP-NPs were synthesized using a dopamine template immobilized on the surface of the beads with a mixture of APTMS and AEAPTMS with a molar ratio of 1:1. Both the fluorescent intensity and radius were sensitive to dopamine but were also sensitive to DOPA, as shown in Figure 6. The fMIP-NPs were synthesized using APTMS and PTMS with a molar ratio of 5:5 (1:1), 3:7, and 1:9. The fluorescent intensity and radius of the fMIP-NP prepared with the 3:7 and 1:9 molar ratios were seen to increase with the dopamine concentration but were almost insensitive to the DOPA concentration. The relative change caused by the addition of 15 μM of dopamine or DOPA is listed in Table 1. The fMIP-NP prepared with the 1:9 ratio was insensitive to DOPA, but the sensitivity was lower than that with the 3:7 ratio. The lower number of imprinted cavities in the fMIP-NP matrix presumably accounts for its lesser sensitivity. The results show that to produce a selective and sensitive fMIP-NP, a solid surface template with a moderate surface density is required. As can be seen in Figure 7, neither dopamine nor DOPA has any effect on the radius or fluorescence intensity of the dopamine-specific NIP-NP. These findings reveal that the fluorescence sensitivity and radius of the fMIP-NP on the dopamine concentration are only due to the dopamine-imprinted cavity, the quality of which is controlled by the surface density of the immobilized template. The lowest detection limit of dopamine using the fMIP-NP was around 1.5 nM. Zhang reported that the dopamine concentrations in the striatum of Parkinson’s disease rats and normal rats are 0.9 and 3 µM, respectively [26]. Thus, the achieved sensitivity of fMIP-NP would be sufficient to detect dopamine in the central nervous systems of animals. 

### 3.3. Sensitivity of fMIP-NP of Acetylcholine

**Figure 8:**
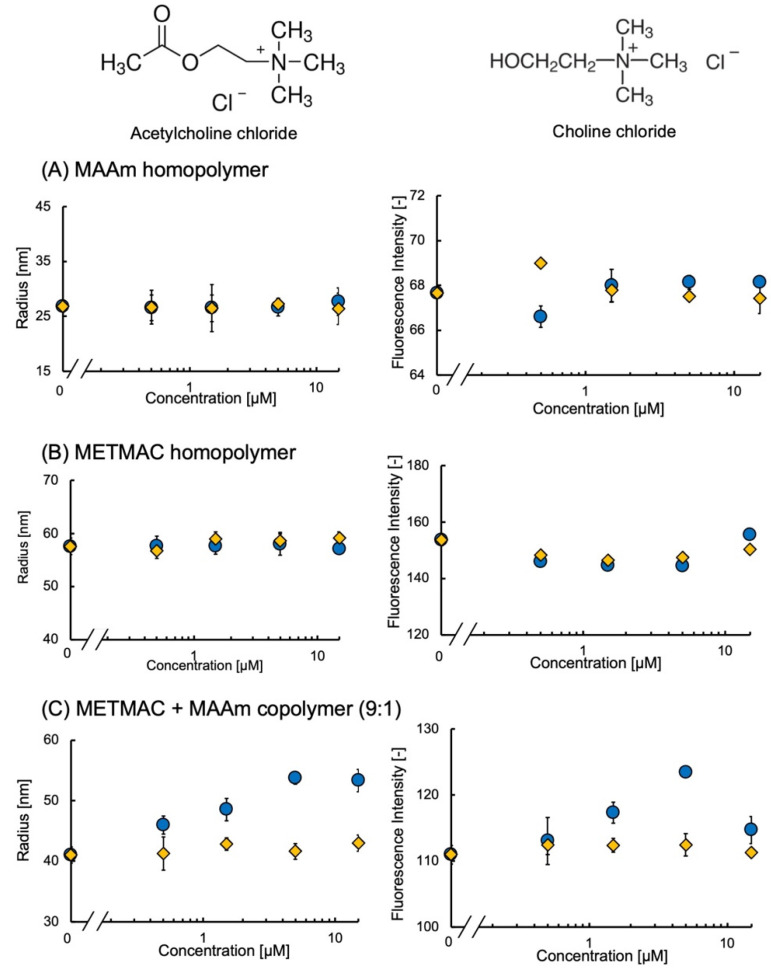
Effect of the acetylcholine (circles) and choline (diamonds) on the radius (**left**) and fluorescent intensity (**right**) of the fNIP-NP synthesized with the (**A**) MAAM homopolymer, (**B**) METMAC homopolymer, and (**C**) copolymer of METMAC and MAAm. (Molar ratio of monomers prepared was 9:1).

The dummy template of acetylcholine was prepared by copolymerizing METMAC and MAAm with varying molar ratio grafting on the surface of glass beads. The fMIP-NP was synthesized by copolymerizing the MAA, EDMA, and DAF in the presence of the dummy template. The dependency of the fluorescent intensity and radius on the concentration of acetylcholine and choline is shown in Figure 8. The fluorescent intensity and radius of the fMIP-NP synthesized with the dummy template prepared with METMAC: MAAm= 0:10 (MAAm homopolymer) and 10:0 (MTEMAC homopolymer) in the molar ratio were insensitive to both acetylcholine and choline. However, the radius and fluorescent intensity of the fMIP-NP prepared with the ratio of 9:1 increased with the increase in acetylcholine concentrations from 0 to 5 µM but were completely insensitive to choline.

The dummy template containing the MAAm homopolymer lacks the trimethylammonium chloride ethyl ester group seen in acetylcholine. Thus, it is obvious that the fluorescent nanoparticles prepared with the MAAm homopolymer were insensitive to acetylcholine or choline. The low sensitivity of fMIP-NP prepared with the METMAC homopolymer is probably due to the poor accessibility of the monomers to the trimethylammonium chloride ethyl ester group. Thus, these results indicate the trimethylammonium chloride ethyl ester group with proper density can function as the dummy template of acetylcholine for the preparation of fMIP-NP with a moderate density (METMAC: MAAm = 9:1). When the radius was saturated from 5 to 15 µM of acetylcholine, the fluorescence intensity dropped. The fluorescence of the fMIP-NP may have been quenched because of the specific binding of the excess acetylcholine. The lowest concentration of the detection would be higher than 1 µM. Several papers have reported acetylcholine concentrations in brain plasma fluid in the tens of pM [27,28,29]. Unfortunately, this fMIP-NP has limited sensitivity to acetylcholine, though the concentrations of acetylcholine around the neurons may be higher. Nevertheless, this result indicates that a dummy template (with a structure similar to that of the target) can be used to synthesize fMIP-NP for templates with structures that are difficult to fix on a glass surface.

### 3.4. Speculation about the Mechanism of Sensing

The experimental data show that fMIP-NP synthesized with the template fixed on the glass surface with moderate surface density expands through the interaction with the target molecules used as a template. They also reveal that, with few exceptions, the swelling is accompanied by an increase in the fluorescence intensity. As the fNIP-NP showed neither swelling nor fluorescence change, these decreases may be attributed to the specific interaction of the imprinted cavity with the target substance.

**Figure 9:**
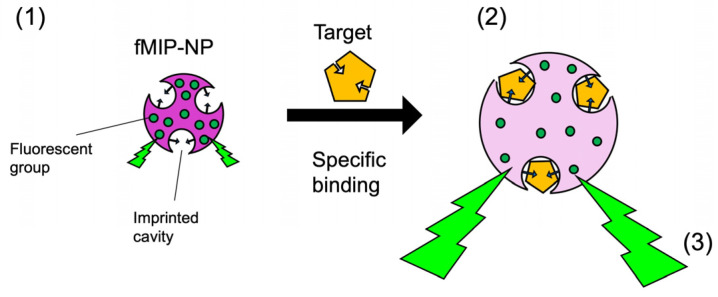
Possible principle of increased fluorescence intensity due to swelling of fMIP-NPs upon specific binding. (1) Fluorescent functional groups are densely packed in fMIP-NPs, and the fluorescence intensity is suppressed by self-quenching. (2) However, the distance between the fluorescent functional groups increases by swelling in conjunction with specific binding to the target substance used as a template. (3) Finally, self-quenching is suppressed, and fluorescence intensity increases.

The specific interactions of the template and MIP are discussed here. The fluorescence intensity and excitation and fluorescence spectra were independent of the fMIP-NP, fMIP-NP, and concentration of the target and analogues; they were almost identical to those of fluorescein. This means that the fluorescence resonance energy transfer was not involved in the change in fluorescence intensity associated with specific binding. Therefore, we are focusing on the self-quenching of fluorescence intensity as shown in Figure 9. High concentrations of fluorescein are self-quenching [30]; the fluorescein groups in fMIP-NP are also thought to be self-quenching. Wang et al. reported a sensing method for copper ion detection using fluoresceine emitted from anionic nanogels, including the fluorescein group. The anionic nanogels contracted due to electrostatic interactions with copper cations, resulting in a decrease in the distance between fluorescein anchored in the nanogel matrix and a decrease in fluorescence due to self-quenching [31]. In this study, the increase in fluorescence intensity due to specific interactions with the template seems to be based on the same principle, as shown in Figure 8. In our previous research, we have found that the nanoparticle of MIP in heparin is swelled by the specific interaction with heparin which has a molecular weight of around 10,000. The results in this work report that the MIP nanoparticle for a small target molecule (MW < 1000) indicates a similar swelling induced by the specific interaction between the imprinted cavity and target molecule. 

This study may have provided a way to expand the targeting of MIP nanoparticles using solid-phase synthesis to those with smaller molecular weights. Solid-phase synthesis has so far focused mainly on high molecular weight (MW > 10,000) materials such as proteins [24,32,33,34,35,36]. Although it has shown high selectivity for targets with molecular weights up to about 1500 [37], there are few reports on targets with molecular weights lower than that, and the selectivity seems to be low [38]. This may be due to the fact that molecules with small molecular weights have few characteristic functional groups, and when immobilized on a solid-phase surface as a template, they show high interaction with each other, and the interaction with the functional monomer is small. There may also be no functional group that can be immobilized under mild conditions. In this study, we showed that MIP nanoparticles with high specificity for small molecule targets can be prepared by the “solid-phase synthesis method” by appropriately blending anchors (aminoalkylsilanes) to adjust the density for immobilization or by graft copolymerizing monomers similar in structure to the target substance with spacer monomers to form a dummy template. The results show that MIP nanoparticles with a high specificity for small molecular targets can be synthesized using the graft copolymerization of a monomer with a similar structure to the target material and a spacer monomer to form a dummy template. This method is simple and will be widely accepted. This study also shows that it is possible to synthesize nanoparticles of MIP that swell with high specificity for the template by optimizing the density at which the template is immobilized. 

The results also indicate that optically self-reporting material for neurotransmitters can be easily made using a simple molecular imprinting method. The detection of the secretion of neurotransmitters is important for understanding neuronal activity in the central nervous system in animals, which is conventionally performed using amperometric and microdialysis methods. The former is based on the detection of the redox current of the target neurotransmitter at a microelectrode implanted in the brain, which is affected by concomitant redox species (e.g., uric acid or ascorbic acid). The latter is based on the collection of neurotransmitters diffusing into the hollow fiber of a semipermeable membrane, which requires several minutes.

Monoamine-type transmitters are easy to immobilize on glass via glutaraldehyde and aminoalkylsilanes, but some kinds of neurotransmitters do not have the proper functional groups to bind with glass surfaces in mild conditions. However, the results of the fMIP synthesized with poly (METMAC-*co*-MAAm), as a dummy template of acetylcholine, indicate that a dummy template is disposable for synthesizing fMIP-NP for the targets that are difficult to immobilize on the glass surface.

In this study, the fluorescence intensity of fMIP-NP enhanced by the neurotransmitter is only 10% at most. It is difficult to accurately measure the concentration of neurotransmitters in the brain with this fMIP-NP. However, an attempt to observe neurotransmitter secretion by combining the voltage imaging method, in which neurons are stained with a voltage-sensitive dye and observed under a fluorescence microscope, is not too ambitious. In the voltage imaging technique, the change in fluorescence intensity corresponding to the generation of neural action potentials is at most 0.1–0.3% [39]. The magnitude of the membrane potential change cannot be accurately measured with this method. However, the imaging can identify the location and timing of significant membrane potential changes (action potentials, excitatory postsynaptic potentials, inhibitory postsynaptic potentials, etc.). These findings from the voltage imaging have actually contributed significantly to the elucidation of neural networks. Therefore, if we can identify the timing and site of transmitter release using a fluorescence microscope for membrane potential imaging to observe neural samples adsorbed or stained with transmitter template fMIP-NPs, it will contribute to the elucidation of neural networks. The fMIP-NP can be prepared via the classical radical polymerization of acrylic monomers, which takes only a few days to synthesize and costs less than USD 10 per batch. In combination with proper fluorescent microscopy and imaging analysis technology, the fMIP-NP would be a potential tool for probing the neurotransmitter secretion of living neurons. However, the development of the method requires a technique to allow the fMIP-NP to be adsorbed at the surface of neurons using proper modification of the fMIP-NPs. We believe that imaging neurotransmitter secretion from living neurons stained with the developed fMIP-NP will contribute to studying the mechanisms of the activity of the central nervous system (e.g., learning, memory, and emotions).

## 4. Conclusions

A nanoparticle of MIP, whose size and fluorescent intensity are sensitive to neurotransmitters with high selectivity, can be synthesized using the transmitter immobilized on the surface of the glass beads with the regulation of its surface density. 

## 5. Patents

We have a patent pending for “Molecularly Imprinted Nanoparticles with Fluorescent Functional Groups (JP2018132527A)”.

## Figures and Tables

**Table 1:** Effect of sensitivity and selectivity of the fluorescent intensity of fMIP-NP using dopamine template immobilized by the blended silane couplers.

Molar Ratio of Anchors	Relative Change [%] in Fluorescence Intensity by		Sensitivity Ratio [-]
	15 μM Dopamine	15 μM DOPA	
APTMS: AEATMS = 5:5	3.6	1.5	2.4
APTMS: PTMS =			
5:5	8.7	2.8	3.1
3:7	7.3	0.1	73
1:9	4.2	0.1	42

## Data Availability

Not applicable.

## References

[1] Siegelbaum S.A., Südhof T.C., Tsien R.W., Kandel E., Koester J.D., Mack S.H., Siegelbaum S.A. (2022). Transmitter release. Principles Neural Science.

[2] Kissinger P.T., Hart J.B., Adams R.N. (1973). Voltammetry in brain tissue—A new neurophysiological measurement. Brain Res..

[3] Adams R.N. (1990). In vivo electrochemical measurements in the CNS. Prog. Neurobiol..

[4] Rodeberg N.T., Sandberg S.G., Johnson J.A., Phillips P.E., Wightman R.M. (2017). Hitchhiker’s Guide to Voltammetry: Acute and Chronic Electrodes for in Vivo Fast-Scan Cyclic Voltammetry. ACS Chem. Neurosci..

[5] Gardier A.M. (2013). Antidepressant activity: Contribution of brain microdialysis in knock-out mice to the understanding of BDNF/5-HT transporter/5-HT autoreceptor interactions. Front. Pharmacol..

[6] van Heesch F., Prins J., Konsman J.P., Korte-Bouws G.A., Westphal K.G., Rybka J., Olivier B., Kraneveld A.D., Korte S.M. (2014). Lipopolysaccharide increases degradation of central monoamines: An in vivo microdialysis study in the nucleus accumbens and medial prefrontal cortex of mice. Eur. J. Pharmacol..

[7] Cudjoe E., Bojko B., de Lannoy I., Saldivia V., Pawliszyn J. (2013). Solid-phase microextraction: A complementary in vivo sampling method to microdialysis. Angew. Chem. Int. Ed. Engl..

[8] Zhang M., Fang C., Smagin G. (2014). Derivatization for the simultaneous LC/MS quantification of multiple neurotransmitters in extracellular fluid from rat brain microdialysis. J. Pharm. Biomed. Anal..

[9] Hou M.L., Lin C.H., Lin L.C., Tsai T.H. (2015). The Drug-Drug Effects of Rhein on the Pharmacokinetics and Pharmacodynamics of Clozapine in Rat Brain Extracellular Fluid by In Vivo Microdialysis. J. Pharmacol. Exp. Ther..

[10] Konig M., Thinnes A., Klein J. (2018). Microdialysis and its use in behavioural studies: Focus on acetylcholine. J. Neurosci. Methods.

[11] Zestos A.G., Luna-Munguia H., Stacey W.C., Kennedy R.T. (2019). Use and Future Prospects of in Vivo Microdialysis for Epilepsy Studies. ACS Chem. Neurosci..

[12] Chen Y., Pu Q., Yu F., Ding X., Sun Y., Guo Q., Shi J., Zhang J., Abliz Z. (2022). Comprehensive quantitative method for neurotransmitters to study the activity of a sedative-hypnotic candidate using microdialysis and LCxLC-MS/MS. Talanta.

[13] Okubo Y., Sekiya H., Namiki S., Sakamoto H., Iinuma S., Yamasaki M., Watanabe M., Hirose K., Iino M. (2010). Imaging extrasynaptic glutamate dynamics in the brain. Proc. Natl. Acad. Sci. USA.

[14] Patriarchi T., Cho J.R., Merten K., Howe M.W., Marley A., Xiong W.-H., Folk R.W., Broussard G.J., Liang R., Jang M.J. (2018). Ultrafast neuronal imaging of dopamine dynamics with designed genetically encoded sensors. Science.

[15] Corre J., van Zessen R., Loureiro M., Patriarchi T., Tian L., Pascoli V., Luscher C. (2018). Dopamine neurons projecting to medial shell of the nucleus accumbens drive heroin reinforcement. Elife.

[16] Augustine V., Ebisu H., Zhao Y., Lee S., Ho B., Mizuno G.O., Tian L., Oka Y. (2019). Temporally and Spatially Distinct Thirst Satiation Signals. Neuron.

[17] Mohebi A., Pettibone J.R., Hamid A.A., Wong J.T., Vinson L.T., Patriarchi T., Tian L., Kennedy R.T., Berke J.D. (2019). Dissociable dopamine dynamics for learning and motivation. Nature.

[18] Jing M., Li Y., Zeng J., Huang P., Skirzewski M., Kljakic O., Peng W., Qian T., Tan K., Zou J. (2020). An optimized acetylcholine sensor for monitoring in vivo cholinergic activity. Nat. Methods.

[19] Patriarchi T., Mohebi A., Sun J., Marley A., Liang R., Dong C., Puhger K., Mizuno G.O., Davis C.M., Wiltgen B. (2020). An expanded palette of dopamine sensors for multiplex imaging in vivo. Nat. Methods.

[20] Sun F., Zhou J., Dai B., Qian T., Zeng J., Li X., Zhuo Y., Zhang Y., Wang Y., Qian C. (2020). Next-generation GRAB sensors for monitoring dopaminergic activity in vivo. Nat. Methods.

[21] Labouesse M.A., Cola R.B., Patriarchi T. (2020). GPCR-Based Dopamine Sensors-A Detailed Guide to Inform Sensor Choice for In vivo Imaging. Int. J. Mol. Sci..

[22] Shea K., Yan M., Roberts M.J. (2002). Molecularly Imprinted Materials-Sensors and Other Devices.

[23] Yoshimi Y., Oino D., Ohira H., Muguruma H., Moczko E., Piletsky S.A. (2019). Size of Heparin-Imprinted Nanoparticles Reflects the Matched Interactions with the Target Molecule. Sensors.

[24] Poma A., Guerreiro A., Whitcombe M.J., Piletska E.V., Turner A.P.F., Piletsky S.A. (2013). Solid-phase synthesis of molecularly imprinted polymer nanoparticles with a reusable template-“Plastic Antibodies”. Adv. Funct. Mater..

[25] Liu Q.-H., Liu J., Guo J.-C., Yan X.-L., Wang D.-H., Chen L., Yan F.-Y., Chen L.-G. (2009). Preparation of polystyrene fluorescent microspheres based on some fluorescent labels. J. Mater. Chem..

[26] Zhang Y., Xu S., Xiao G., Song Y., Gao F., Wang M., Zhao H., Xing G., Cai X. (2019). High frequency stimulation of subthalamic nucleus synchronously modulates primary motor cortex and caudate putamen based on dopamine concentration and electrophysiology activities using microelectrode arrays in Parkinson’s disease rats. Sens. Actuators B Chem..

[27] Yamada H., Otsuka M., Fujimoto K., Kawashima K., Yoshida M. (1996). Determination of acetylcholine concentration in cerebrospinal fluid of patients with neurologic diseases. Acta Neurol. Scand..

[28] Togashi H., Matsumoto M., Yoshioka M., Hirokami M., Tochihara M., Saito H. (1994). Acetylcholine Measurement of Cerebrospinal Fluid by In Vivo Microdialysis in Freely Moving Rats. Jpn. J. Pharmacol..

[29] Nirogi R., Mudigonda K., Kandikere V., Ponnamaneni R. (2010). Quantification of acetylcholine, an essential neurotransmitter, in brain microdialysis samples by liquid chromatography mass spectrometry. Biomed. Chromatogr..

[30] Lakowicz J.R. (2006). Principles of Fluorescence Spectroscopy.

[31] Wang F., Planalp R.P., Seitz W.R. (2019). A Cu (II) Indicator Platform Based on Cu (II) Induced Swelling that Changes the Extent of Fluorescein Self-Quenching. Polymers.

[32] Ambrosini S., Beyazit S., Haupt K., Bui B.T.S. (2013). Solid-phase synthesis of molecularly imprinted nanoparticles for protein recognition. Chem. Commun..

[33] Xu J.J., Medina-Rangel P.X., Haupt K., Bui B.T.S. (2017). Guide to the Preparation of Molecularly Imprinted Polymer Nanoparticles for Protein Recognition by Solid-Phase Synthesis. Methods Enzymol..

[34] Poma A., Guerreiro A., Caygill S., Moczko E., Piletsky S. (2014). Automatic reactor for solid-phase synthesis of molecularly imprinted polymeric nanoparticles (MIP NPs) in water. RSC Adv..

[35] Cowen T., Stefanucci E., Piletska E., Marrazza G., Canfarotta F., Piletsky S.A. (2020). Synthetic Mechanism of Molecular Imprinting at the Solid Phase. Macromolecules.

[36] Cavalera S., Chiarello M., Di Nardo F., Anfossi L., Baggiani C. (2021). Effect of experimental conditions on the binding abilities of ciprofloxacin-imprinted nanoparticles prepared by solid-phase synthesis. React. Funct. Polym..

[37] Mazzotta E., Turco A., Chianella I., Guerreiro A., Piletsky S.A., Malitesta C. (2016). Solid-phase synthesis of electroactive nanoparticles of molecularly imprinted polymers. A novel platform for indirect electrochemical sensing applications. Sens. Actuators B Chem..

[38] Chen L., Muhammad T., Yakup B., Piletsky S.A. (2017). New immobilisation protocol for the template used in solid-phase synthesis of MIP nanoparticles. Appl. Surf. Sci..

[39] Loew L., Canepari M., Zecevic D. (2011). Design and Use of Organic Voltage Sensitive Dyes. Membrane Potential Imaging in the Nervous System.

